# Bioengineering and Stem Cell Technology in the Treatment of Congenital Heart Disease

**DOI:** 10.3390/jcm4040768

**Published:** 2015-04-22

**Authors:** Alexis Bosman, Michael J. Edel, Gillian Blue, Rodney J. Dilley, Richard P. Harvey, David S. Winlaw

**Affiliations:** 1 Victor Chang Cardiac Research Institute, Lowy Packer Building, 405 Liverpool St., Darlinghurst NSW 2010, Australia; E-Mails: a.bosman@victorchang.edu.au (A.B.); r.harvey@victorchang.edu.au (R.P.H.); 2 St. Vincent’s Clinical School and School of Biotechnology and Biomolecular Sciences, University of New South Wales, Kensington NSW 2052, Australia; E-Mail: edel.michael@gmail.com; 3 Control of Pluripotency Laboratory, Department of Physiological Sciences I, Faculty of Medicine, University of Barcelona, Hospital Clinic, Casanova 143, 08036 Barcelona, Spain; 4 Senior Research Fellow, Sydney Medical School, University of Sydney, Sydney NSW 2006, Australia; 5 School of Anatomy Physiology & Human Biology and The Harry Perkins Institute for Medical Research (CCTRM), Senior Research Fellow at the University of Western Australia, Crawley WA 6009, Australia; 6 International Research Fellow, Victor Chang Cardiac Research Institute, Darlinghurst NSW 2010, Australia; 7 Heart Centre for Children, The Children’s Hospital at Westmead, Westmead NSW 2145, Australia; E-Mail: Gillian.Blue@health.nsw.gov.au; 8 Sydney Medical School, University of Sydney, Sydney NSW 2006, Australia; 9 Ear Science Institute Australia, Centre for Cell Therapy and Regenerative Medicine and School of Surgery, University of Western Australia, Nedlands WA 6009, Australia; E-Mail: rodney.dilley@earscience.org.au

**Keywords:** congenital heart disease, hypoplastic left heart, inducible pluripotential stem cells, bioengineered myocardium

## Abstract

Congenital heart disease places a significant burden on the individual, family and community despite significant advances in our understanding of aetiology and treatment. Early research in ischaemic heart disease has paved the way for stem cell technology and bioengineering, which promises to improve both structural and functional aspects of disease. Stem cell therapy has demonstrated significant improvements in cardiac function in adults with ischaemic heart disease. This finding, together with promising case studies in the paediatric setting, demonstrates the potential for this treatment in congenital heart disease. Furthermore, induced pluripotent stems cell technology, provides a unique opportunity to address aetiological, as well as therapeutic, aspects of disease.

## 1. Clinical Consideration of Congenital Heart Disease

Treatment of congenital heart disease (CHD) occupies a unique place in the human history of cardiovascular medicine. This dates back to the pioneering development of early heart-lung machines in the early 1950s. Subsequent development of this technology allowed correction of simple heart defects in childhood that would have otherwise led to early death, with further evolution permitting routine adult cardiac surgery for ischaemic and valvular heart disease, now accepted as “everyday surgery”.

In modern CHD clinical research, both patients and practitioners look forward to similar paradigm shifts in treatments to address some of the inadequacies of current management that continue to impact individuals, families and workplaces. There are now more adults with congenital heart disease than children in advanced societies [1] and whilst many are effectively “cured” with childhood intervention (such as closure of infant ventricular septal defects) others have an ongoing need for close medical management including those with single ventricle physiology [2] or who require repeated surgeries, for example, those who will need replacement of right ventricle to pulmonary artery conduits.

The burden of disease is significant and has physical, psychological and economic impacts [3]. CHD occurs in ~7–8 in 1000 live births [4,5]. A subset of CHD is invariably lethal around birth unless treated, and these cases present significant challenges with respect to surgical reconstruction, critical care patient management, long term follow up and the ethics of focusing major health resources onto few individuals. CHD successfully treated in childhood carries a strong likelihood of complications in later life and a life-long emotional and financial burden for affected families [6]. The dramatic reduction in mortality after surgical correction of CHD in recent years has been accompanied by increasing recognition of poor neurological outcomes in survivors of CHD, which may involve genetic factors, abnormal brain perfusion and development *in utero* and/or susceptibilities to hypoxia resulting from CHD, or other environmental parameters such as anesthesia [7,8]. A key bottleneck in patient care is the transition from childhood to adulthood, where patients may be lost to follow up.

Childhood treatment is very costly and paediatric cardiac surgery is the most common reason for admission to paediatric intensive care. Over the last three decades, surgery has become more complex and is generally performed earlier—often during the neonatal period—to gain better functional outcomes in the long term. A diagnosis of CHD is associated with important psychosocial dysfunction with many parents reporting symptoms equivalent to post-traumatic stress disorders, high levels of parental depression and ongoing anxiety with similar problems observed in adolescent and adult survivors [9].

Addressing causation of CHD has been a high priority over the last decades, particularly for the minority of cases that show familial inheritance. Classical linkage analysis has been the mainstay methodology underpinning these studies. Studies on the interaction between genetic and environmental factors have revealed clinically important perturbations of the highly conserved and tightly regulated developmental cardiogenic processes but only in a smaller number of patients with single gene disorders and associated syndromes [10]. In the new era of genetic research, genome wide association studies have identified areas of common chromosomal variation associated with the most common but simple form of CHD, secundum ASD [11], but with relatively low odds ratios and limited clinical application. Massively parallel sequencing of the whole exome [12] and its more targeted approaches [13] have dramatically accelerated the disease gene discovery pipeline, yielding answers for additional families. Polygenic contribution, variable penetrance and variation in phenotype present ongoing challenges.

On the horizon is a new era of stem cell-based therapies and bioengineering, and it is hoped that these approaches can help reduce the burden of CHD. In broad terms, stem cell and bioengineering approaches may make contributions to: (i) improving structural solutions in repair of malformed hearts; (ii) improving the function of repaired hearts and their circulation; and (iii) facilitating modelling of CHD to advance our understanding of its molecular underpinnings. These will be discussed further below.

### 1.1. Structural Solutions

In paediatric heart surgery, there is a need to address the current demands of the circulation as well as future growth. Many forms of advanced neonatal surgery involve utilisation of the existing ventriculo-arterial connection as the systemic outflow (usually through a large ventricular septal defect) and creation of an extra-anatomic right ventricle to pulmonary artery conduit. Repairs of pulmonary atresia with VSD, and truncus arteriosus are examples that utilise this approach. Usually either a human cadaveric allograft (homograft) is used for this purpose, or a bovine jugular venous conduit, combining a “tube” with a valve. A larger group of patients, those with tetralogy of Fallot, may require pulmonary valve replacement, currently also utilising allograft or xenograft tissue valves.

Whilst effective in the short term, the long term functional outcomes of such approaches are poor, with all requiring replacement within 3 to 8 years depending on the size of the patient, patient growth, host response to the allograft or xenograft and other factors including the occasional development of endocarditis. Supplies of both types of conduit are limited and are associated with significant expense. Allosensitisation to donated human products can also be a problem if transplantation is later required. Percutaneous approaches are now available that are suitable for some patients, particularly in the adolescent group, but as xenoproducts they remain susceptible to immune mediated structural valve deterioration and infection.

Many biologic approaches have been attempted to improve longevity of the implanted valve, including decellularising and re-seeding allograft tissue with host endothelial cells [14]; however this approach has not yet been shown to produce meaningfully increased graft survival or somatic growth [15]. Generation of a vascularised matrix that can then be seeded and shaped [16] is emerging as an approach that avoids the need for allograft material but will require complex 3D construction to simulate tube and valve formation. Patients undergoing the Fontan operation as a final step in construction of a cavo-pulmonary connection have been managed with tissue engineered vascular grafts to convey the inferior vena caval blood to the pulmonary arteries [17]. This is valuable proof of principle work yielding understandings of optimal matrix construction, albeit that no significant growth is presently required of this connection using current surgical approaches [18]. Electrospinning and microfabrication techniques to engineer scaffolds that support the growth of valvular interstitial cells and mesenchymal stem cells [19] offer a way to customise the size and shape of the replacement tissue, perhaps guided by 3D imaging of the planned recipient. Repopulation with engineered patient-specific cells utilising adult stem cell or induced pluripotent stem cell technologies would seem logical for the future [20,21].

### 1.2. Stem Cells to Improve Cardiac Function

There is extensive and ongoing work to support the use of stem cells in recovery from myocardial infarction in adult populations, particularly using bone marrow derived cells, albeit that the rationale for such studies is under intense scrutiny [22]. Regeneration of scar tissue into functional myocardium and improved ventricular performance are the aims of such interventions with recent promise [23,24,25]. In paediatric cardiology the aim would be the optimisation of ventricular performance for children subjected to volume or pressure loads, usually after correction of the structural abnormalities that promote ventricular dysfunction. There is particular interest in the subpopulation of patients with a functional single ventricle, especially those who have undergone complex single ventricle surgery such as the Norwood operation for hypoplastic left heart (HLH) [26].

Typically HLH patients would be infants after the first two stages of surgery involving long periods of cardiopulmonary bypass and shorter periods of planned and “protected” myocardial ischaemia. An increased volume load related to the shunt providing pulmonary blood flow after the initial operation adds to the work that the single right ventricle must perform, which is already at an anatomic disadvantage being a morphologic right ventricle working against systemic vascular resistance. It is not uncommon for the function of such ventricles to deteriorate, particularly after second stage surgery, promoting atrioventricular valve regurgitation which positively reinforces the ventricular dysfunction. Relative coronary insufficiency [27] or a primary myocardial process may contribute. Structural abnormalities have been identified in single right ventricular tissue [28]. Ventricular performance is a major determinant of suitability for the last stage of the single ventricle pathway, Fontan completion (total cavo-pulmonary connection) as well as performance and survival with the Fontan circulation.

In parallel with studies in animal models [29,30], various approaches to ventricular support using stem cell technology are being trialled in CHD patients with differing donor cell origins and modes of administration, as outlined by Tarui *et al.* [31]. A number of stem cell populations have been described in the mammalian heart using cell surface markers and various functional assays including colony formation, and growth and differentiation potential *in vitro* and *in vivo* [22] Cardiosphere-derived cells are among the first populations to be trialled in humans for ischaemic heart disease in adults [32,33]. They are heterogeneous cell preparations derived from the 3D cellular clusters (cardiospheres) that can be readily established from heart biopsies, and which are thought to provide a harbour (niche) for cells with stem or progenitor cell properties during *in vitro* culture. Cells derived from atrial tissue and administered via the intracoronary route at cardiac catheterisation, have been trialled in patients with HLH in Phase I and Phase II clinical trials, with other groups utilising umbilical cord [34] and bone marrow derived cell fractions [31]. Phase 1 trials have indicated the safety of this approach with some improvement in right ventricular systolic function evident, and Phase 2 studies are underway. In the recently reported Phase 1 study of autologous cardiosphere-derived cells delivered via the intracoronary route [35], no safety concerns were raised and an improvement in right ventricular function was observed at 18 months compared to controls. The effect size is encouraging and clinically relevant (a 10% increase in right ventricular ejection fraction). The use of autologous cells represents a clear advantage in this environment. Similar approaches may be of benefit in paediatric heart failure presenting as dilated cardiomyopathy.

Uncertainty persists about the mechanism by which the stem cells might induce functional improvement. In ischaemic disease and cardiomyopathy, paracrine activation of local regenerative pathways may significantly contribute to the improvements in performance, while tissue replacement due to stem cell deployment does not seem to be a dominant feature in animal studies [36].

Cord blood stem cells have been shown to engraft and augment right ventricular function in an ovine model in the presence of increased workload [30]. A similar model of right ventricular overloading in rats demonstrated improved diastolic dysfunction and suppression of ventricular fibrosis following skeletal myoblast transplantation (Hoashi *et al.* 2009). Case reports demonstrate improvement in ventricular function following intracoronary delivery of bone marrow derived cells in children with terminal cardiomyopathy [37,38] as well as ventricular failure following surgery for HLH [39]. In HLH, adaptation of the right ventricle to increased work load may require cellular proliferation beyond the capability of the intrinisic regenerative systems. The capacity for autologous modified cells in CHD to influence cardiac performance or myocyte proliferation may be diminished by the persistence of genetic characteristics that caused or contributed to abnormal development during primary cardiogenesis. However, the development of refined cell therapy approaches may support the growth and development required.

## 2. Induced Pluripotent Stem Cells to Study Causation in Congenital Heart Disease

**Figure 1:**
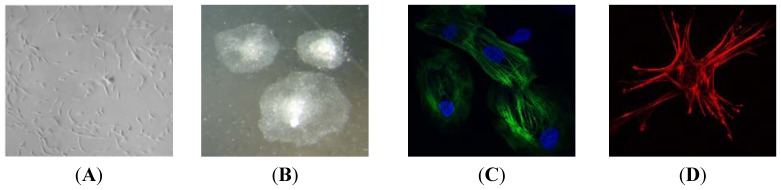
(**A**) Patient-derived fibroblasts generated from a skin biopsy; (**B**) Undifferentiated iPSC colonies derived from patient-derived fibroblasts; (**C**) Cardiomyocytes derived from iPSC stained for the sarcomeric protein, cardiac troponin T; (**D**) Smooth muscle cells derived from iPSC stained for the cell scaffolding protein, alpha smooth muscle actin.

Induced pluripotent stem cells (iPSC) can be created from virtually any somatic cell, most commonly from dermal fibroblasts [40]. These pluripotent cell types are created by the reprogramming of adult cells to a pluripotent state, giving them the ability to differentiate into all cell types of the human body, including cardiomyocytes (see Figure 1) as well as smooth muscle, endothelial and epicardial cells, the highly specialised cell types of the heart. This makes iPSC an invaluable resource for the study of CHD. The technology offers the unique opportunity to create human models of disease and development in a patient-specific context that incorporates the individual clinical features of the disease. Additionally, iPSC provide material to study the earliest time points in development, previously difficult due to restrictions on the availability of primary human tissue for study.

iPSC are playing an increasing role in personalised medicine, specifically in disease profiling of both rare and common diseases, and in the design of personalised therapies. Due to the recent success of directed differentiation protocols [41,42,43], iPSC allow the provision of lineage-specific stem and progenitor cells, as well as differentiated specialised cell types, for disease research, cellular therapies and tissue engineering. However, before iPSC are used as a source of biologic material for clinical application, concerns regarding the oncogenic effect of retained transgenes [44] and trans-differentiation need to be addressed [45]. Until then, iPSC are being increasingly used as a test bed to study development and disease mechanism. In the cardiac area, iPSC approaches have been successful in assessing the functional disorder associated with LEOPARD Syndrome [46,47] and various arrhythmias and cardiomyopathies [48,49].

iPSC are playing an increasing role in personalised medicine, specifically in disease profiling of both rare and common diseases, and in the design of personalised therapies. Due to the recent success of directed differentiation protocols [41,42,43], iPSC allow the provision of lineage-specific stem and progenitor cells, as well as differentiated specialised cell types, for disease research, cellular therapies and tissue engineering. However, before iPSC are used as a source of biologic material for clinical application, concerns regarding the oncogenic effect of retained transgenes [44] and trans-differentiation need to be addressed [45]. Until then, iPSC are being increasingly used as a test bed to study development and disease mechanism. In the cardiac area, iPSC approaches have been successful in assessing the functional disorder associated with LEOPARD Syndrome [46,47] and various arrhythmias and cardiomyopathies [48,49].

The approach is applicable to CHD particularly for cell-autonomous genetic disorders affecting, for example, the development or function of cardiomyocytes that can be modelled in 2D cell cultures or 3D tissue constructs [50,51]. The approach has its obvious limitations with respect to modelling the complex tissue interactions necessary for organ structure, and the non-cell autonomous environmental or epigenetic influences on disease. However, rapid progress is being made on directed differentiation of highly complex organoids and tissue layers from pluripotent stem cells [52,53], opening up vast new potential for therapies and modelling disease in this system.

Using a patient-specific *in vitro* model of HLH is of particular interest to clinicians and scientists in the field attempting to reconcile the most common theory about the genesis of HLH—reduced transventricular flow and altered loading during development—with the heterogeneity in morphology as well as performance and decline observed in clinical cases [54,55]. An iPSC approach will complement the forward genetic approach being taken in mice [56]. While it has been suggested that HLH is essentially a severe form of valve malformation [56,57], some cases of HLH have a bulky LV and small but formed mitral and aortic valves, whilst others have barely a recognisable LV cavity. In combination these studies lead to speculation that a primary myocardial disorder is present in HLH, which likely predetermines the size and function of the ventricle and perhaps contributes to difficulties in later childhood in some with this condition. HLH is thought to have a high genetic component with complex inheritance, and is often associated with chromosomal abnormalities [58], which could impact on either valvular structures or ventricular cardiomyocyte growth and function, or both. Of the limited number of gene pathways implicated in HLH [58], the transcription factors NKX2-5 and NOTCH1 are known to be involved in both valvular and chamber development [59,60]. Both genes are also involved in aortic coarctation and bicuspid aortic valve, which exist within the spectrum of left-sided abnormalities that includes HLH at its most severe end [61,62,63].

Jiang and colleagues made iPSC from a single HLH patient and used them to derive cardiomyocytes by directed differentiation. They found a number of important primary cardiac defects including altered expression of key cardiac transcription factors, fewer beating clusters and reduced myofibrillar organisation, persistence of a fetal gene expression pattern as well as altered calcium transients and calcium handling [54]. Kobayashi *et al.* analysed single clones from three HLH patients, using a clone from a patient with bicuspid aortic valve and total anomalous pulmonary venous connection as a control [55]. They showed reduced expression of a number of cardiac transcription factors at late time points after induced cardiomyocyte differentiation, and associated changes in total chromatin marks—di-methylation on histone H3 lysine 4, tri-methylation on histone H3 lysine 27, and acetylation of histone H3. Whether the reported changes are common to all cases of HLH remains to be seen. Such molecular phenotypes in patient specific iPSC-derived cardiomyocytes raises the possibility that disease modelling using the iPSC platform can provide both molecular diagnosis, as has been utilised in other cardiovascular diseases [64] and cell therapy into the future.

### Bioengineering Heart Muscle Using iPS Cells

Investigations into the creation of functional heart tissue *in vitro* by tissue engineering techniques using donor cardiomyocytes is still in its very early stages [65,66,67,68]. While there are no clinical applications of the method to date, cardiac tissue engineering has seen progress over the last twenty years in all four of the elements central to this method: generation of donor cardiomyocytes, development of scaffold materials and control of cell survival, engraftment and growth with bioactive molecules (see recent reviews [51,69,70]). The latest developments include *ex vivo* and *in vivo* approaches that promote the growth of vascular and structural elements of cardiac tissue [71,72]. Growing cells as sheets has made possible the insertion of iPSC-derived cardiomyocytes into the porcine heart for short term benefits [73]. Human embryonic stem cell-derived cardiomyocytes have been successfully engrafted in a non-human primate model of myocardial infarction [74]. This approach included development and application of mass culture techniques able to support production and delivery of a billion cells, selection of delivery techniques to optimise survival, such as a supportive hydrogel scaffold and application of a cocktail of preconditioning regimes. While this demonstrates potential for successful remuscularization of the human heart, issues with the incomplete maturation of cardiomyocytes, as well as arrhythmogenesis, need to be addressed. Contractile and vascularised human cardiac tissues have also been created from iPS cells [75,76] to provide long term survival and contractility, and 3D microtissues derived from iPSC also show promise for transplantation [77].

The ability to make whole functional hearts or bioengineered patches and conduits is challenging and has not been achieved for clinical use thus far. A form of bioengineered hearts have been configured using human iPSC-derived multipotential cardiovascular progenitors (MCP), which are likely similar to the earliest cardiac progenitors in heart development, by implanting them into a decellularized donor mouse heart [78]. The decellularized heart provides an excellent 3D structure for bioengineering whole organs or surgical implants as it utilises the natural extracellular matrix to promote cardiomyocyte proliferation, differentiation and function. The use of such native cardiac scaffold provides appropriate cues for engraftment, promotes rapid vascularisation and also avoids the biocompatibility problems of some artificial scaffold materials. MCP may offer an advantageous cell type for cardiac tissue bioengineering applications as they can potentially self-organise into structures containing cardiomyocytes, smooth muscle cells and endothelial cells, guided by extracellular matrix cues. However, before a whole heart can be bioengineered, a number of challenges remain, including safeguards surrounding the use of iPSC as discussed, as well as modulation of the immune response and, in CHD applications, finding ways that allow growth of the graft along with the patient’s heart.

## 3. Conclusions

Emerging technology in stem cells and bio-engineering may address major issues in congenital heart disease that limit lifespan and reduce quality of life for a significant number of children and adults. iPSC technology offers an opportunity to provide both molecular diagnosis and, in the future, tissue based therapy for some of the more complex reconstructive tasks in congenital heart disease.
